# Supplementary material for: MIF is a 3’ flap nuclease that facilitates DNA replication and promotes tumor growth
Source: Nat Commun. 2021 May 19;12:2954. doi: 10.1038/s41467-021-23264-z (PMC8134555; doi:10.1038/s41467-021-23264-z)
Supplement: Supplementary file 1 — Supplementary Information [file 41467_2021_23264_MOESM1_ESM.pdf]

Supplemental Information

Table 1. Mass spectrometry analysis of MIF protein preparations

Accession	Description	Coverage (%)	Spectrum counts			
			Buffer	Sample 1	Sample 2	Sample 3
**P14174**	**Macrophage migration inhibitory factor**	**42**	**0**	**127**	**346**	**985**
P0A6Y8	Chaperone protein DnaK	89	0	15	99	26
P0C8J8	D-tagatose-1,6-bisphosphate aldolase subunit GatZ	75	0	5	66	27
Q8WXH0	Nesprin-2	0	0	5	12	4
P0A6F5	60 kDa chaperonin	85	0	0	56	8

Table 2.

Oligonucleotide Sequences of MIF Substrates, Templates, sgRNA targets and others primers

Name	Sequence
DS-7nt	Forward: 5'-CTCAGCCTCCCAAGTAGCTGACAGCACGAG-3'
	Reverse: 5'-CTCGTGCTGTCAGCTACTTGGGATTACAGG-3'
DS-4nt	Forward: 5'-CTCAGCCTCCCAAGTAGCTGACAGCACGAG-3'
	Reverse: 5'-CTCGTGCTGTCAGCTACTTGGGATTAC-3'
DS-3nt	Forward: 5'-CTCAGCCTCCCAAGTAGCTGACAGCACGAG-3'
	Reverse: 5'-CTCGTGCTGTCAGCTACTTGGGATTA-3'
DS-2nt	Forward: 5'-CTCAGCCTCCCAAGTAGCTGACAGCACGAG-3'
	Reverse: 5'-CTCGTGCTGTCAGCTACTTGGGATT-3'
DS-1nt	Forward: 5'-CTCAGCCTCCCAAGTAGCTGACAGCACGAG-3'
	Reverse: 5'-CTCGTGCTGTCAGCTACTTGGGAT-3'
DS-0nt	Forward: 5'-CTCAGCCTCCCAAGTAGCTGACAGCACGAG-3'
	Reverse: 5'-CTCGTGCTGTCAGCTACTTGGGA-3'
EL-F	5'-AATGATACGGCGACCACCGAGATCTACACTCTTTCCCTACACGACGCTCTTCCGA TCTCCCAACCTGCTTTATATATCTTGTGGAAAGGACGAAACACC-3'
EL-R0	5'-GGTGTTTCGTCCTTTCCA-3'
EL-R1	5'-GGTGTTTCGTCCTTTCCAG-3'
EL-R2	5'-GGTGTTTCGTCCTTTCCAGT-3'
EL-R3	5'-GGTGTTTCGTCCTTTCCAGTT-3'
hMIFsgRNA1	5'-GAGGAACCCGTCCGGCACGG-3'
hMIFsgRNA2	5'-AGCTCGGAGAGGAACCCGTC-3'
hMIFsgRNA3	5'-CATCGCGGTGCACGTGGTCC-3'
POLD1F	5'-ATCGAGTGCGCCGGCCGCAA-3'
hMIFshRNA1	5'-TGCTGTTGACAGTGAGCGCTCATCGTAAACACCAACGTGCTAGTGAAGCCACAGA TGTAGCACGTTGGTGTTTACGATGAATGCCTACTGCCTCGGA-3'
hMIFshRNA2	5'-TGCTGTTGACAGTGAGCGACGCGCAGAACCGCTCCTACAGTAGTGAAGCCACAG ATGTACTGTAGGAGCGGTTCTGCGCGCTGCCTACTGCCTCGGA-3'

hMIFshRNA3	5'-TGCTGTTGACAGTGAGCGAAGGGTCTACATCAACTATTACTAGTGAAGCCACAGA TGTAGTAATAGTTGATGTAGACCCTGTGCCTACTGCCTCGGA-3'
M13 Seq	5'-GACAGGTTTCCCGACTGGAAAGC-3'
PARP1 FL (Full-length)	Forward: 5'-CCGCTCGAGGCCACCATGGGTAAGCCTATCCCTAACCCTCTCCTCGGT CTCGATTCTACGGCGGAGTCTTCGGA-3'
	Reverse: 5'-CGTCTAGATTACCACAGGGAGGT-3'
∆Zn1-3	Forward: 5'-CCGCTCGAGGCCACCATGGGTAAGCCTATCCCTAACCCTCTCCTCGGT CTCGATTCTACGGCCTCGGCTCCTGCTGCTGT-3'
	Reverse: 5'-CGTCTAGATTACCACAGGGAGGT-3'
∆Zn1-BRCT	Forward: 5-CCGCTCGAGGCCACCATGGGTAAGCCTATCCCTAACCCTCTCCTCGGT CTCGATTCTACGGCCCCAAGAGGGAAGT-3'
	Reverse: CGTCTAGATTACCACAGGGAGGT
∆WGR-CAT	Forward: 5'-CCGCTCGAGGCCACCATGGGTAAGCCTATCCCTAACCCTCTCCTCGGT CTCGATTCTACGGCGGAGTCTTCGGA-3'
	Reverse: 5'-CGTCTAGA GTTGATACCTTCCTCCTT-3'
∆CAT	Forward: 5'-CCGGATCCGCCACCATGGGTAAGCCTATCCCTAACCCTCTCCTCGGT CTCGATTCTACGGCGGAGTCTTCGGA-3'
	Reverse: 5'-CGTCTAGACTACTTGGTGCCAGGAT-3'

Table 3. KEY RESOURCES TABLE

REAGENT or RESOURCE	Source	IDENTIFIER
Antibodies		
anti-MIF, (1: 1,000)	Abcam	Cat #ab36146; #ab175189
anti-PCNA, (1: 1,000)	Santa Cruz Biotechnology	Cat #sc-56
anti-Actin, (1: 5,000)	Proteintech	Cat #66009-1
anti-FLAG, (1: 5,000)	Sigma-Aldrich	Cat #F3165
anti-PARP1, (1:1,000)	Proteintech	Cat #22999-1
anti-PAR, (1: 1,000)	Trevigen	Cat #4336-BPC-100
anti-BrdU, (1: 250)	Invitrogen	Cat #MA3-071
anti-BudU (CldU, 1: 250)	Abcam	Cat #ab6326
anti-BrdU (IdU, 1: 50)	BD Biosciences	Cat #347580
anti-γH2AX, (1: 1,000)	Millipore	Cat #05636
anti-H2AX, (1: 1,000)	Proteintech	Cat #10856-1-ap
anti-53BP1, (1:250)	Thermo Fisher Scientific	Cat #APPA6566
Normal rabbit IgG antibody, (2.5 μg/ml)	Cell Signaling Technology	Cat #2729S
Normal mouse IgG antibody, (2.5 μg/ml)	Santa Cruz Biotechnology	Cat #sc-2025
HRP-conjugated goat anti-Rabbit IgG, (1:5000)	Jackson ImmunoReaserch	Cat #NC9611376
HRP-conjugated goat anti-Mouse IgG, (1:5000)	Jackson ImmunoReaserch	Cat #NC9491974
HRP-conjugated Donkey anti-Goat IgG, (1:5000)	Jackson ImmunoReaserch	Cat #705035147
Alexa Fluor 568 Donkey anti-mouse IgG(H+L), (1:1000)	Invitrogen	Cat #A10337
Alexa Fluor 488 Donkey anti-mouse IgG(H+L), (1:1000)	Invitrogen	Cat #A21206
CY2 affinipure donkey anti-rat, (1:1000)	Jackson ImmunoReaserch	Cat #NC1113744
Chemicals, reagents		
Thymidine	Sigma	T1895
BrdU	Sigma	19160
EdU	Invitrogen	A10044

Streptavidin agrose	EMD Millipore	692033
CldU	Sigma	C6891
IdU	Sigma	I7135
Click-it EdU Alexa Fluor 488 Imaging Kit	Invitrogen	C10337
Biotin Azide	Invitrogen	B10184
Colcemid	Sigma	234109-M
Chemiluminescent Nucleic Acid Detection Module	Thermo Fisher Scientific	692033

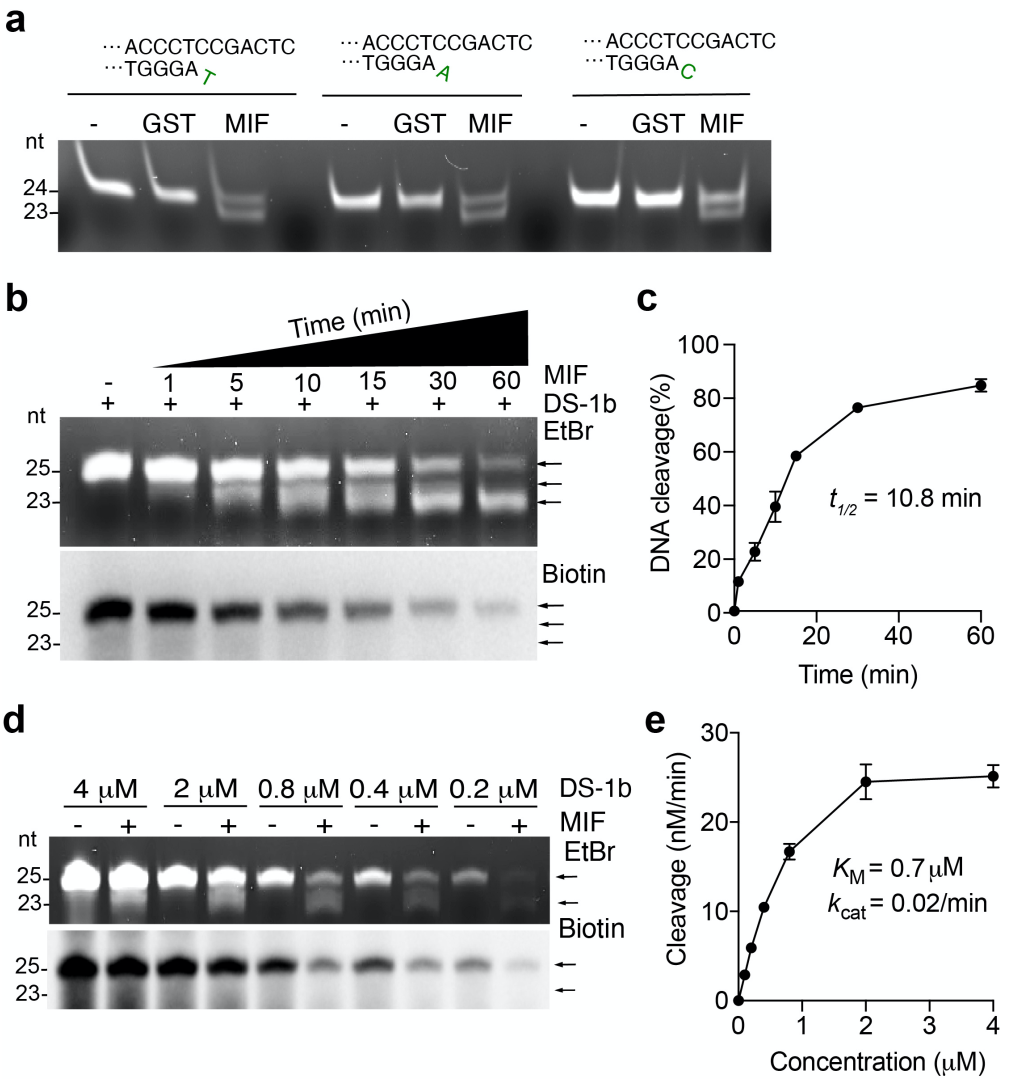

Supplementary Fig. 1. MIF cleaves 3' biotin-labelled Y-shaped dsDNA (DS-1b) in a time- and concentration-dependent manner.

a MIF cleaves away 3' unpaired nucleotides independent of its sequence.

b, c MIF (2 μM) cleaves 3' biotin-labeled dsDNA substrate DS-1b (0.8 μM) in a time-dependent manner. Representative images from three independent experiments are shown in **b**. Data are quantified in **c** (mean ± SEM, *n* = 3 biologically independent experiments).

d, e MIF (2 μM) cleaves 3' biotin-labeled dsDNA substrate DS-1b in a concentration-dependent manner. Representative images from three independent experiments are shown in **d**. Data are quantified in **e** (mean ± SEM, *n* = 3 biologically independent experiments).

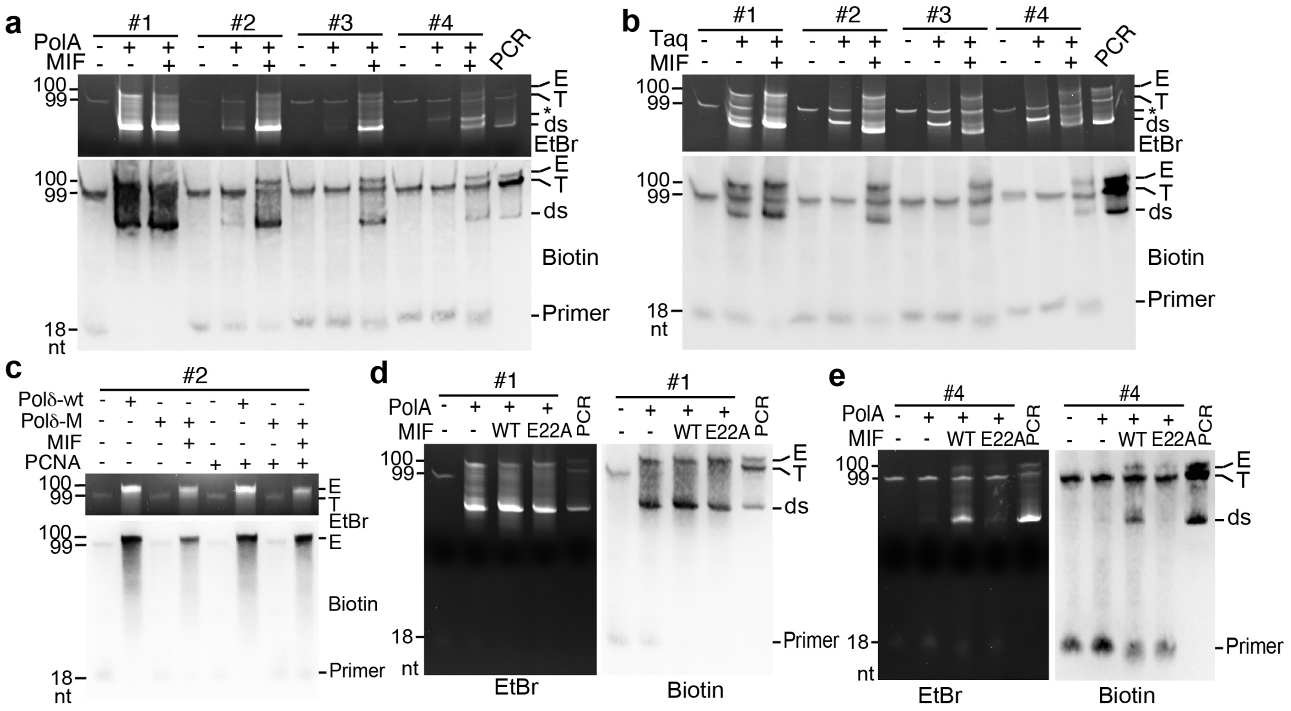

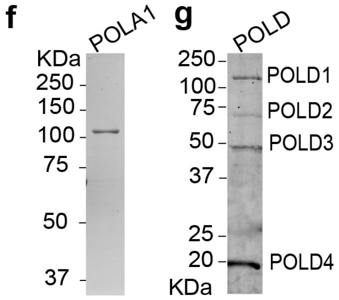

h

Polymerase	Total Plaques	Mutant Plaques	Mutant Frequency
Taq	2717	6	22 / 10^4
Taq + GST	3496	5	14 / 10^4
Taq + MIF-WT	5481	4	7 / 10^4
Taq + MIF-E22A	2720	6	22 / 10^4

i

Taq

Clones	Position	From	To	Notes
Taq #1	105	G	T	mutation
	169	AA	AGA	insertion
Taq #2	175	G	C	mutation
	245	TT	CC	mutation
Taq #3	45	AG	ACG	insertion
	62	A	T	mutation
Taq #4	253	C	T	mutation
Taq #5	164	G	C	mutation
Taq #6	170	AT	TA	mutation

Taq + GST

Clones	Position	From	To	Notes
Taq+GST#1	232	C	A	mutation
	258	A	G	mutation
	260	C	G	mutation
Taq+GST#2	286	GA	GCA	insertion
	296	AGG	TTT	mutation
	300	CG	TT	mutation
Taq+GST#3	104	G	T	mutation
Taq+GST#4	327	GC	GTC	insertion
	394	TT	TGT	insertion
Taq+GST#5	104	G	T	mutation

Taq + MIF

Clones	Position	From	To	Notes
Taq+MIF #1	77	TGG	TG	deletion
Taq+MIF #2	77	TGG	TG	deletion
Taq+MIF #3	77	TGG	TG	deletion
Taq+MIF #4	77	TGG	TG	deletion

Taq + E22A

Clones	Position	From	To	Notes
Taq+E22a#1	277	C	T	mutation
	285	C	T	mutation
Taq+E22a#2	242	TGG	TG	deletion
	251	C	G	mutation
	297	G	A	mutation
	313	G	C	mutation
	332	T	A	mutation
	343	G	C	mutation
Taq+E22a#3	98	GT	GAT	insertion
Taq+E22a#4	97	CG	AA	mutation
Taq+E22a#5	29	T	C	mutation
	33	C	G	mutation
	50	C	G	mutation
	80	CC	GCC	insertion
	95	G	T	mutation
	98	G	C	mutation
Taq+E22a#6	45	AG	GA	mutation
	49	T	A	mutation
	59	G	A	mutation
	73	GC	TT	mutation
	82	T	G	mutation
	84	GT	AA	mutation

Supplementary Fig. 2. MIF coordinates with low fidelity DNA polymerase to facilitate DNA elongation.

a, b *In vitro* DNA elongation assay mediated by 3' nuclease-deficient polymerase α (Polα) (**a**) or Taq DNA polymerase (**b**) using a 99-bp DNA template and primers listed in Fig.2a in the presence or absence of MIF. DNA was visualized by EtBr staining and Biotin immunoblot. A regular PCR DNA serves as the positive control. “E” and “ds” represent the ss and ds elongation product, respectively. T indicates the complex of the 99-bp DNA template and biotin-labeled primer. The star (*) indicates the intermediate elongation product.

c *In vitro* DNA elongation assay mediated by Pol δ or 3' nuclease-deficient Pol δ D402A mutant (Polδ-M) (5 ng/μl) in the presence or absence of MIF (2 μM) or PCNA (7.5 ng/ul) using a 99-bp DNA template (0.4 μM) and primers (0.4 μM) listed in Fig.2a. E represents the elongation product. T indicates the complex of the 99-bp DNA template and biotin-labeled primer.

d, e Effects of WT and E22A MIF on Polα-mediated *in vitro* DNA elongation using a 99-bp DNA template and #1 (**d**) or #4 (**e**) primer. DNA was visualized by EtBr staining or Biotin immunoblot.

f, g Purified Pol α and Pol δ proteins shown by Coomassie blue staining.

h, Mutation frequency analysis during gap filling by Taq DNA polymerase in the presence of GST, WT MIF, or E22A MIF proteins.

i Mutation sequence analysis of M13mp18 gap filling mediated by 3' nuclease-deficient Taq DNA polymerase in the presence of GST, MIF or E22A-MIF by Sanger DNA sequencing.

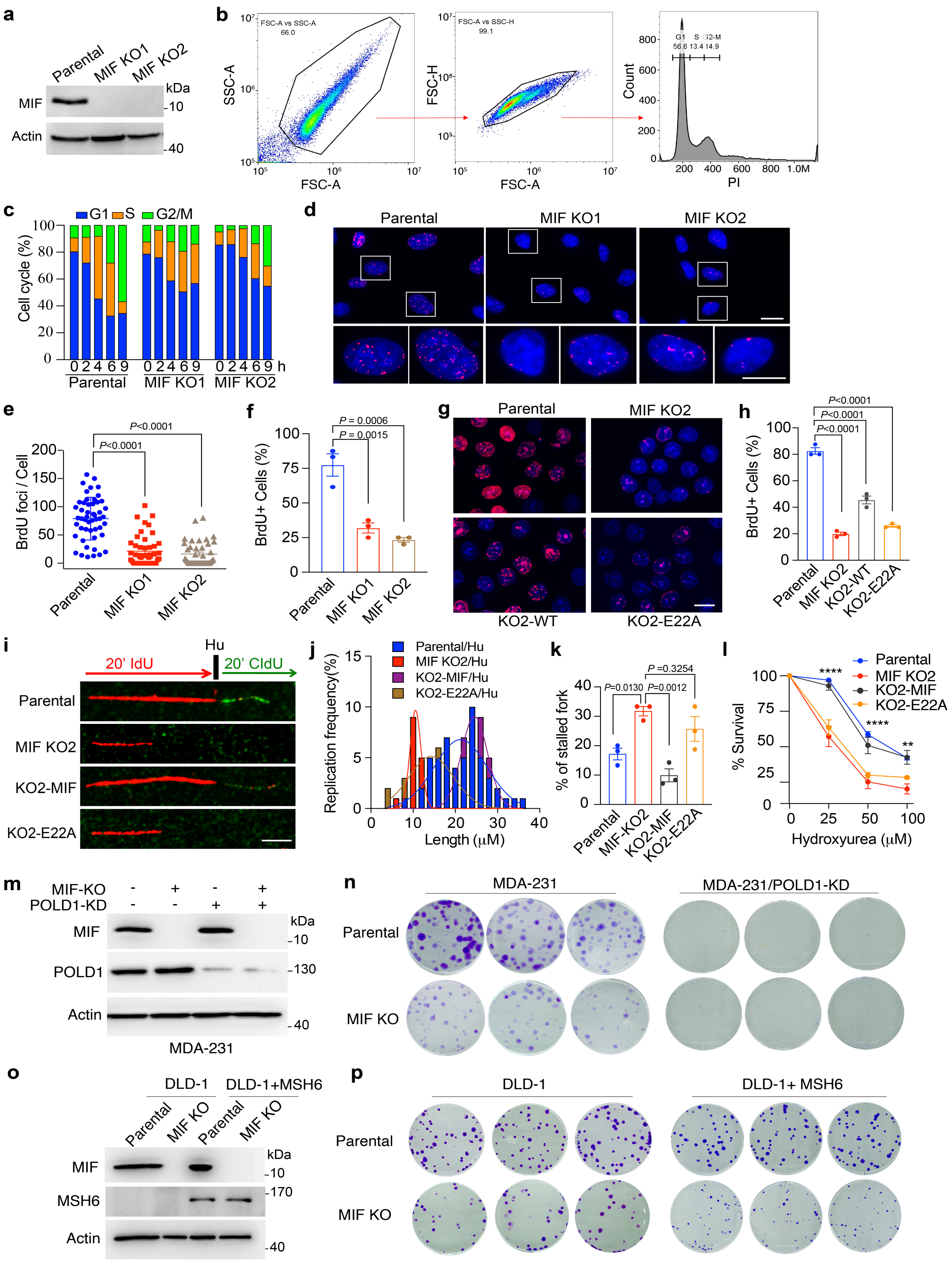

Supplementary Fig. 3. MIF depletion impairs DNA replication.

a Immunoblot analysis of MIF and actin in parental and MIF KO1 or KO2 MDA-MB-231 cells. Representative blots from three independent experiments are shown.

b Flow cytometry gating strategy for MDA-MB-231 cell cycle analysis. First, cell debris was excluded from living cells by forward scatter (FCS-A) vs side scatter (SSC-A) plot. Then single cells were selected within the population in FSC-A vs FSC-H plot. Remaining cells were then applied to Propidium Iodide (PI) vs cell count histogram plot to identify G1, S and G2/M phase based on DNA content.

c Cell cycle distribution analysis by flow cytometry in parental, MIF-KO1 and MIF-KO2 MDA-MB-231 cells following double thymidine synchronization.

d Representative images of BrdU (red) staining in parental, MIF-KO1 and KO2 MDA-MB-231 cells after double thymidine synchronization. Scale bar, 20 μm.

e, f The number of BrdU foci per cell (**e**) and the percentage of BrdU-positive cells (**f**) were quantified. 50 cells per group were analyzed. Cell with > 20 foci were counted as BrdU-positive cells. **** $p < 0.0001$, by one-way ANOVA Dunnett's multiple comparisons test.

g, h Representative images of BrdU (red) staining in parental, MIF-KO2, KO2-MIF and KO2-E22A HCT116 cells after double thymidine synchronization (**g**). Data were quantified in **h** (mean ± SEM, $n = 50$ cells in each group were analyzed over three independent experiments). Scale bar, 20 μm. Cells with > 20 foci were counted as BrdU-positive cells. Statistical significance was determined by one-way ANOVA Dunnett's multiple comparisons test.

i Representative images of stretched DNA fibers in parental MDA-MB-231, MIF-KO2, KO2-WT and KO2-E22A cells, which were incubated with IdU (25 μM) for 20 min, and then treated with hydroxyurea (Hu, 2 mM) for 2 h followed by CIdU (250 μM) incubation for 20 min. Scale bar, 10 μm.

j-k Distribution of the track lengths of CIdU and fork progression are indicated in different groups. $n = 52$ fibers in Parental/Hu group; $n = 30$ fibers in MIF KO2/Hu group; $n = 40$ fibers in KO2-MIF/Hu group and $n = 33$ fibers in KO2-E22A group were measured over three independent experiments. Percentage of stalled fork was calculated in **k** (mean ± SEM, $n = 3$ biologically independent experiments). Statistical significance was determined by one-way ANOVA Dunnett's multiple comparisons test.

l Colony survival rate of parental MDA-MB-231, MIF KO2, KO2-WT and KO2-E22A cells treated without or with hydroxyurea (25-100 μM). Data were presented as mean ± SEM. *n* = 3 biologically independent experiments. **** $p < 0.0001$ for MIF KO2 vs parental and MIF KO2 vs KO2-MIF at Hydroxyurea 25 μM and 50 μM; ** $p < 0.01$ for MIF KO2 vs parental and MIF KO2 vs KO2-MIF at Hydroxyurea 100 μM, by two-way ANOVA Tukey's multiple comparisons test.
m Establishment of MIF KO, POLD1 KD and POLD1 KD/MIF KO MDA-MB-231 cells.
n Colony survival of MIF KO, POLD1 KD and POLD1 KD/MIF KO MDA-MB-231 cells.
o Establishment of MIF KO in DLD1 cells and DLD1+MSH6 cells.
p Colony survival of MIF WT and MIF KO DLD1 and DLD1+MSH6 cells.

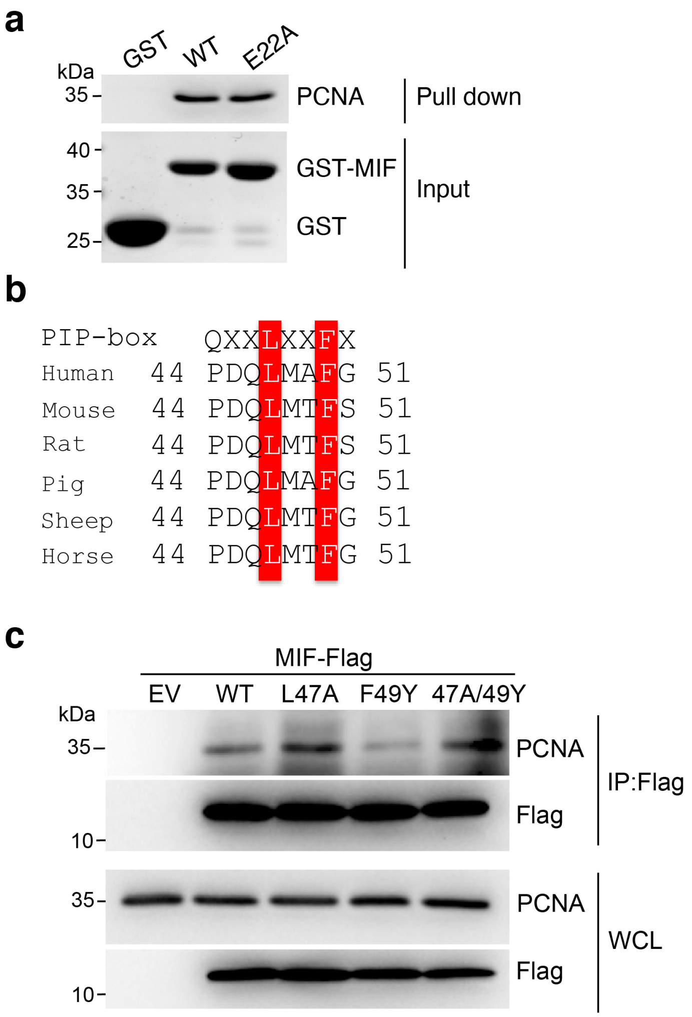

Supplementary Fig. 4. MIF interacts with DNA replication protein PCNA.

a GST-tagged WT MIF and E22A MIF mutant interacts with endogenous PCNA in vitro. Representative blots from three independent experiments are shown.

b Amino acid sequence alignment of PIP-box like domain of MIF across difference species.

c Co-IP of MIF and PCNA in MDA-MB-231 cells. WCL, whole cell lysate.

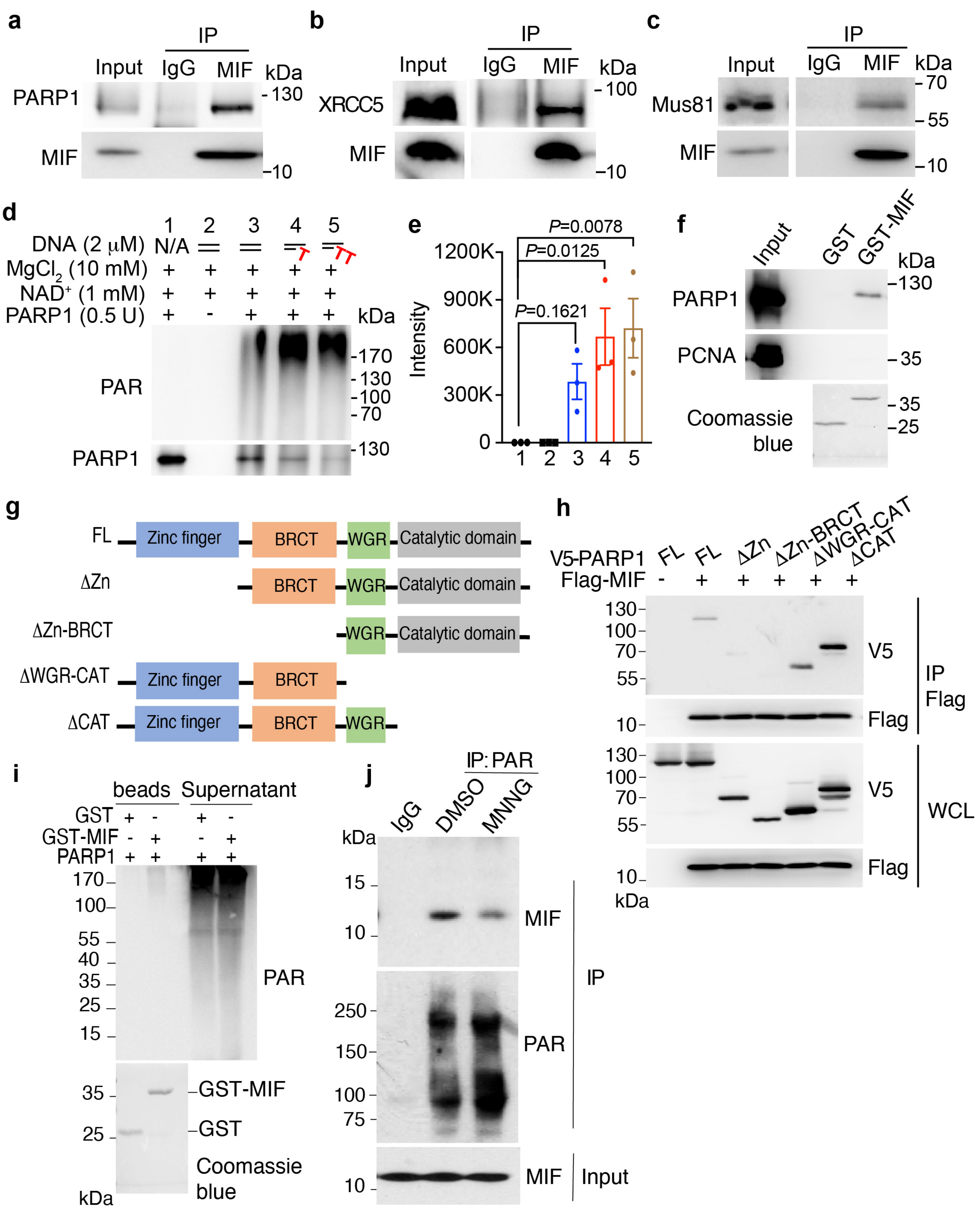

Supplementary Fig. 5. MIF interacts with PARP1 and DNA damage repair proteins.

a Co-IP of endogenous MIF with PARP-1 in MDA-MB-231 cells. Representative blots from three independent experiments are shown.

b Co-IP of endogenous MIF with XRCC5 in MDA-MB-231 cells. Representative blots from three independent experiments are shown.

c Co-IP of endogenous MIF with Mus81 in MDA-MB-231 cells. Representative blots from three independent experiments are shown.

d *In vitro* PARylation assay by incubating PARP1 with dsDNA containing 3' mismatched nucleotides in the presence of 1 mM NAD^+ and 10 mM $MgCl_2$.

e Quantification of PARylation normalized by total intensity of PAR and PARP1 on the same blots. Data were presented as mean ± SEM, n = 3 biologically independent experiments. Statistical significance was determined by one-way ANOVA Dunnett's multiple comparisons test.

f GST-MIF pulled down PARP1, but not PCNA.

g, Scheme of PARP1 domains.

h Domain mapping of PARP1 interaction with MIF by co-IP of PARP1 truncates and Flag-tagged MIF in HEK293 cells.

i In vitro PARylation assay of purified GST-MIF by incubating with 1 U PARP1 protein, 1x activated DNA and 500 μM NAD^+.

j Co-IP of MIF with PAR antibody in HeLa cells with or without MNNG (50 μM, 15 min) treatment.

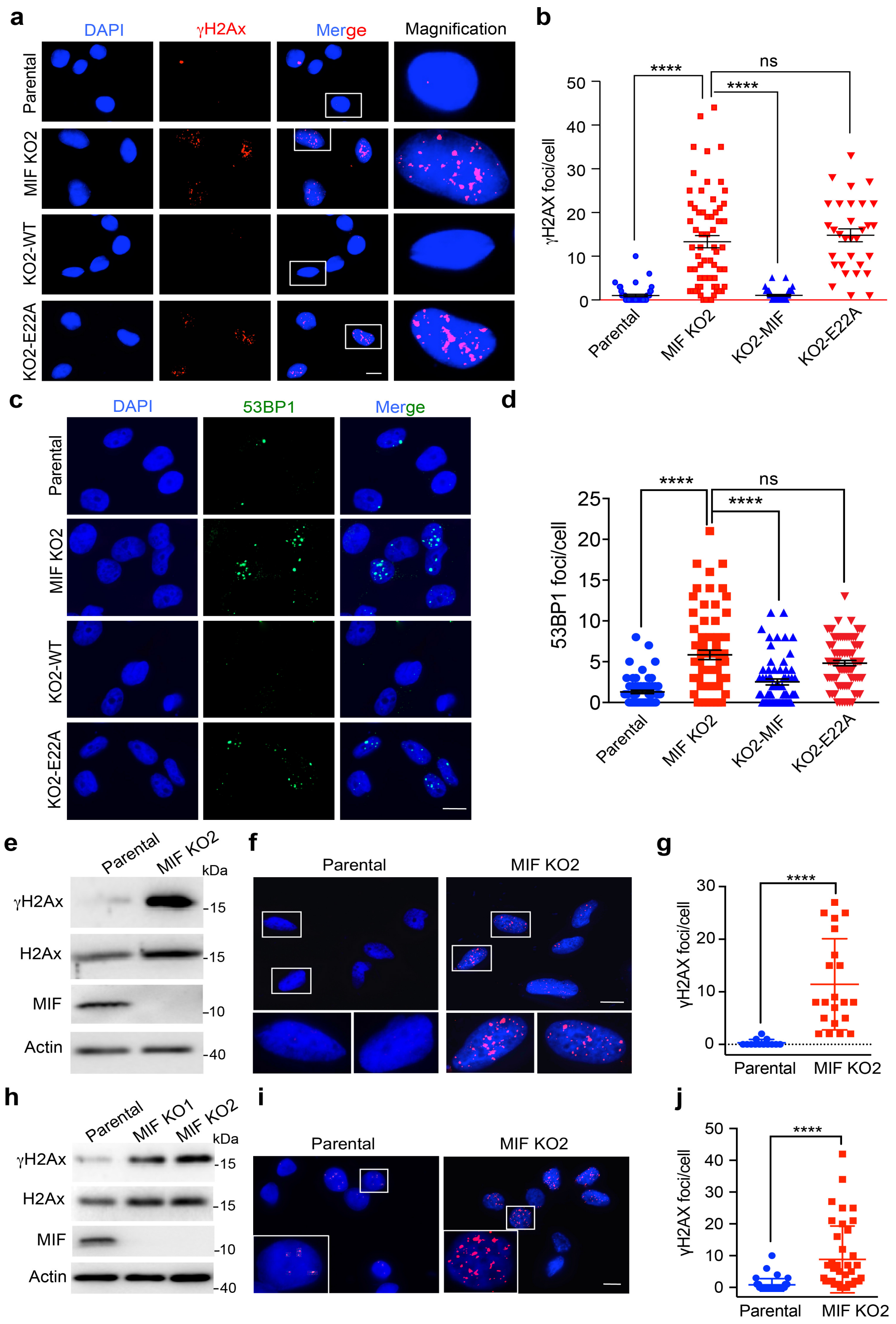

Supplementary Fig. 6. MIF deletion increased the basal levels of DNA damage in cancer cells.

a, b Representative images of γH_2AX foci staining in parental, MIF-KO2, KO2-MIF-WT and KO2-E22A MDA-MB-231 cells (**a**). Data were quantified in **b** (mean ± SEM, n = 3 biologically independent experiments). Scale bar, 20 μm. **** $p < 0.0001$, by one-way ANOVA Dunnett's multiple comparisons test. ns, not significant.

c, d Representative images of 53BP1 foci staining in parental, MIF-KO2, KO2-MIF, and KO2-E22A MDA-MB-231 cells (**c**). Data were quantified in **d** (mean ± SEM, n = 3 biologically independent experiments). Scale bar, 20 μm. **** $p < 0.0001$, by one-way ANOVA Dunnett's multiple comparisons test. ns, not significant,

e γH_2AX protein levels are increased in MIF KO2 LN229 cells. Representative blots from three independent experiments are shown.

f, g Representative images of γH_2AX foci staining in parental and MIF KO2 LN229 cells (**f**). Data were quantified in **g** (mean ± SEM, n = 3 biologically independent experiments). Scale bar, 20 μm. **** $p < 0.0001$ by two-tailed Student's *t* test.

h γH_2AX protein levels are increased in MIF KO1 and KO2 HCT116 cells. Representative blots from three independent experiments are shown.

i, j Representative images of γH_2AX foci staining in parental and MIF KO2 HCT116 cells (**i**). Data were quantified in j (mean ± SEM, n = 3 biologically independent experiments). Scale bar, 20 μm. **** $p < 0.0001$, by two-tailed Student's *t* test.

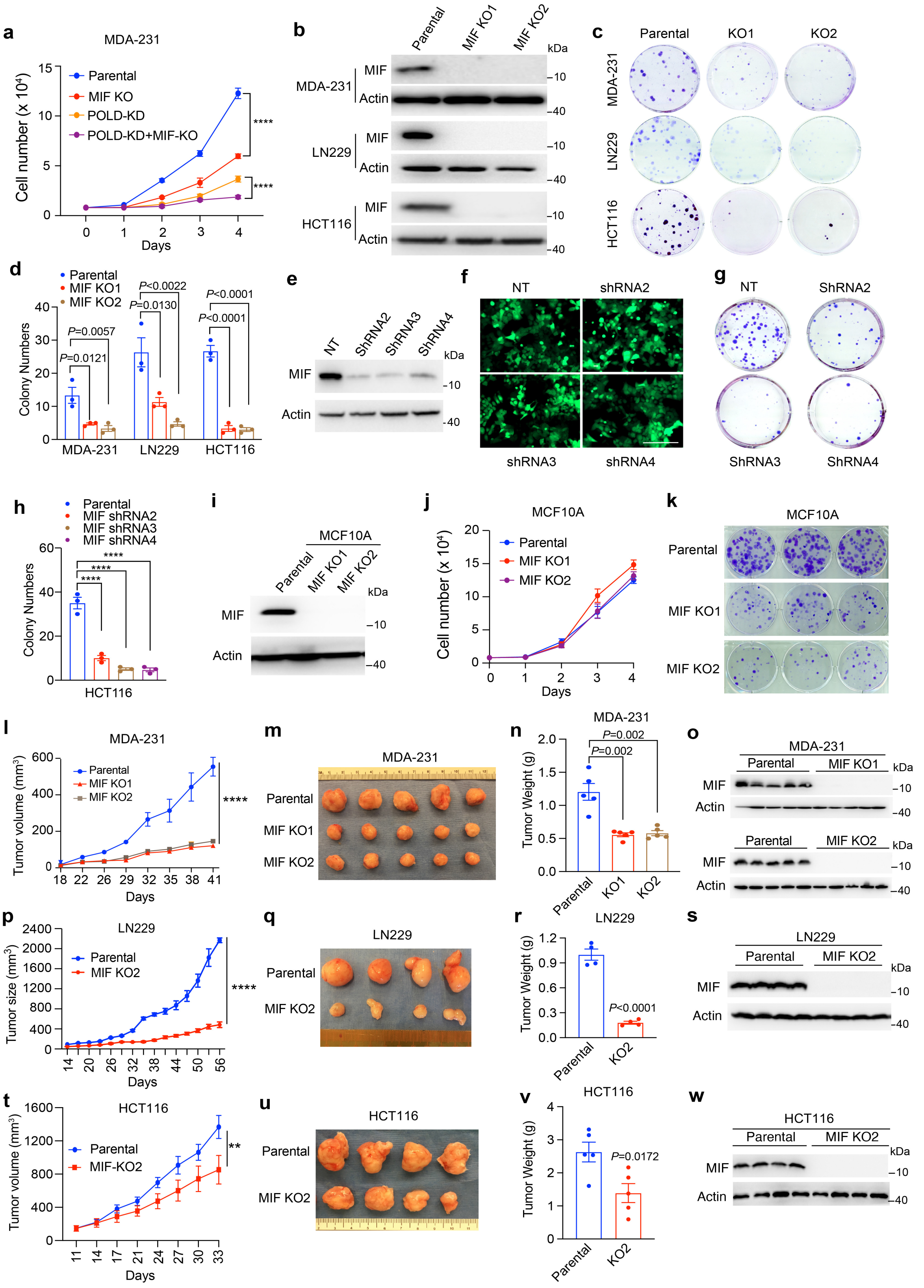

Supplementary Fig. 7. Loss of MIF suppresses cancer cell growth *in vitro and in vivo*.

a Cell growth curve of parental, MIF KO2, POLD KD and POLD KD/MIF KO MDA-MB-231 (MDA-231) cells (mean ± SEM, *n* = 3 biologically independent experiments). **** p < 0.0001 for parental vs MIF KO2, POLD KD vs POLD KD/MIF KO, by two-way ANOVA Tukey's multiple comparisons test.

b Immunoblot analysis of MIF and actin in parental, MIF KO1, and MIF KO2 MDA-MB-231, LN229 and HCT116 cells. Representative blots from three independent experiments are shown.

c, d Colony survival of parental, MIF KO1, and MIF KO2 of MDA-MB-231, LN229 and HCT116 cells. Representative images from three independent experiments were shown in **c** and quantification of colony numbers was shown in **d** (mean ± SEM, *n* = 3 biologically independent experiments). Statistical significance was determined by one-way ANOVA Dunnett's multiple comparisons test.

e Immunoblot analysis of MIF and actin in non-targeting control (NT) and MIF KD HCT116 cells. Representative blots from three independent experiments are shown.

f Representative GFP images showing MIF KD in HCT116 cells. Scale bar, 100 μm.

g, h Colony survival of NT and MIF KD HCT116 cells. Representative images from three experiments were shown in **g** and quantification of colony numbers was shown in **h** (mean ± SEM, *n* = 3 biologically independent experiments). ****P < 0.0001 vs parental by one-way ANOVA Dunnett's multiple comparisons test.

i Immunoblot analysis of MIF and actin in parental, MIF KO1, and MIF KO2 MCF10A cells.

j Cell growth curve of parental, MIF KO1 and MIF KO2 MCF10A cells (mean ± SEM, *n* = 3 biologically independent experiments).

k Colony survival of parental, MIF KO1 and MIF KO2 MCF10A cells.

l-o Growth of parental, MIF KO1, and MIF KO2 MDA-MB-231 tumors in mice. Tumor volume was shown in **l** (mean ± SEM, *n* = 5 mice per group). **** P < 0.0001 versus parental, by two-way ANOVA Tukey's multiple comparisons test. The image of tumors harvested at the end of time point is shown in **m**. Tumor weight was shown in **n** (mean ± SEM, *n* = 5 mice per group). ****P < 0.0001 for MIF KO1 and KO2 vs parental (**l**). Statistical significance was determined by one-way ANOVA Dunnett's multiple comparisons test (**l**, **n**). MIF knockout efficiency in tumor tissues was confirmed by immunoblot (**o**).

p-s Growth of parental and MIF KO2 LN229 tumors in mice. Tumor volume was shown in **p** (mean ± SEM, *n* = 4 mice per group). **** p < 0.0001 versus parental by two-way ANOVA Tukey's multiple comparisons test. The image of tumors harvested at the end of time point is shown in **q**. Tumor weight was shown in **r** (mean ± SEM, *n* = 4 mice per group). **** p < 0.0001, by two-way ANOVA Tukey's multiple comparisons test (**p**) and two-tailed Student's *t* test (**r**). MIF knockout efficiency in tumor tissues was confirmed by immunoblot (**s**). P < 0.0001 parental vs MIF KO2 (**p**), P < 0.0001 parental vs MIF KO2 (**r**).

t-w Growth of parental and MIF KO2 HCT116 tumors in mice. Tumor volume was shown in **t** (mean ± SEM, *n* = 4 mice per group). **** p < 0.0001 versus parental by two-way ANOVA Tukey's multiple comparisons test. The image of tumors harvested at the end of time point is shown in **u**. Tumor weight was shown in **v** (mean ± SEM, *n* = 4 mice per group). Statistical significance was determined by two-tailed Student's *t* test. MIF knockout efficiency in tumor tissues was confirmed by immunoblot (**w**).
